# Supplementary material for: Simple statistical identification and removal of contaminant sequences in marker-gene and metagenomics data
Source: Microbiome. 2018 Dec 17;6:226. doi: 10.1186/s40168-018-0605-2 (PMC6298009; doi:10.1186/s40168-018-0605-2)
Supplement: Supplementary file 1 — Supplementary Appendix: Supplemental Methods. Figure S1. Concentration of contaminant features across oral samples. Figure S2. Histogram of decontam scores in the oral mucosa dataset, weighted by ASV relative abundance. Figure S3. Histogram of decontam scores in the oral mucosa dataset, stratified by reference-based classification. Figure S4. Histogram of decontam scores in the preterm birth dataset. Figure S5. Density plot of scores assigned by the frequency and prevalence methods in the preterm birth dataset. (PDF 1208 kb) [file 40168_2018_605_MOESM1_ESM.pdf]

# Supplemental Information

## Supplemental Methods

**Oral Analysis Study Population**:

Three healthy adults contributed specimens to this study as participants in the healthy control arm of the Oral Microbial Communities in States of Hyposalivation project (NIDCR Grant Number: DE023113-01A1) (also see ref. 37). The study was approved by the Administrative Panels on Human Subjects Research (Stanford IRB protocol #21586), and by the Human Research Protection Program at UCSF (UCSF IRB protocol ##11-06283). Specimens were collected between August and December 2014. All research subjects provided written informed consent prior to specimen collection.

<u>Exclusion criteria</u>:

Study subjects were excluded if they had taken oral antibiotics or antifungals within six months prior to screening date, if they used any medications other than oral contraceptives on a regular basis, if they were under the age of 18, if they had evidence of active oral disease, or if they were unable to adhere to the home- and clinic-collected sampling procedures.

**Sampling procedures**:

Individuals self-collected oral mucosal specimens every day for 30 days using Catch-All™ Sample Collection Swabs (Epicentre Biotechnologies, Madison, WI, USA). The seven sampling sites included: anterior dorsal surface of the tongue, left and right buccal surfaces adjacent to the first molars, upper and lower inner lips adjacent to the central incisors, the middle of the hard palate, and the floor of the mouth adjacent to the attachment site of the lingual frenulum. Subjects were instructed to sample each site by swabbing the area for 10 seconds while applying moderate pressure and moving the swab in a circular motion, to collect specimens at the same time of day, and to store the specimens in their home freezer immediately after collecting the final specimen. Subjects were cautioned to prevent the swab from touching any surface other than the intended sampling site, and to use a new, unopened swab if the swab touches a non-target surface. Subjects also traveled to the Oral Medicine Faculty Clinic in the University of California – San Francisco (UCSF) Dental Center for weekly appointments. There, a UCSF dental clinician collected the same set of seven mucosal specimens following the same protocol. Subjects were instructed to transport their home-collected specimens to the clinic appointments

in weekly batches using provided ice packs and freezer tote bags. Specimens were placed and stored on dry ice immediately following collection. Home- and clinic-collected specimens were then transported to the laboratory where they were stored at -80° C until time of DNA extraction.

**Detailed sample processing procedure**

Specimens were loaded into either a well of a 96-well plate or a 2mL Eppendorf tube, both provided in the PowerSoil® kits described below. Swabs were either clipped into wells 1-2 centimeters above the foam head using a pair of sterile scissors, or bent until broken into Eppendorf tubes. Whole genomic DNA was extracted from each specimen using the PowerSoil®-HTP 96 well Soil DNA Isolation Kit (MO BIO Laboratories, Carlsbad, CA, USA) or the PowerSoil® DNA Isolation Kit (2mL tube-based protocol) according to manufacturer's instructions with two modifications: 1) inclusion of a 10-minute incubation step at 65° C following addition of Solution C1 and 2) final elution in 125μL Solution C6. Genomic DNA was PCR amplified using Golay error-correcting barcoded primers targeting the V4 hypervariable region of the bacterial 16S rRNA gene [51]. The forward PCR primer (5′ AAT GAT ACG GCG ACC ACC GAG ATC TAC ACG CTN NNN NNN NNN NNT ATG GTA ATT GTG TGY CAG CMG CCG CGG TAA 3′) contains (from 5' to 3') an Illumina adapter, a unique 12-nucleotide error-correcting Golay barcode (designated by 'N's), a forward primer pad, a 2-nucleotide forward primer linker, and a broad-range bacterial primer, 515F. The reverse primer (5′ CAA GCA GAA GAC GGC ATA CGA GAT AGT CAG CCA GCC GGA CTA CNV GGG TWT CTA AT 3′) consists of an Illumina adapter, a reverse primer pad, a 2-nucleotide reverse primer linker, and the broad-range bacterial primer 806R.

Each specimen was PCR-amplified according to the following 75-μL PCR reaction set-up and PCR cycling conditions. Replication ranged from 2-4 75-μL replicate reactions per specimen (see sample mapping file for replicates per sample) depending on amplification efficiency, as assayed by presence of a DNA band on agarose gel.

| **Component** | **1 rxn (µL)** |
|---|---|
| Molecular biology grade water (Sigma) | 26.7 |
| 2.5X HotMasterMix (5 PRIME, Gaithersburg, MD, USA) | 30 |
| 100 uM 806R primer (IDT, Coralville, IA, USA) | 0.3 |
| 10 uM 515F primer (barcoded) | 3 |
| Template DNA | 15 |
| **Total (µL)** | 75 |

**Thermal cycling:**

94°C 3 minutes

30 cycles of:

- 94°C 45 seconds
- 52°C 60 seconds
- 72°C 90 seconds

72°C 10 minutes

4°C HOLD

Following confirmation of PCR amplicon product by agarose gel electrophoresis, amplicons were purified using the UltraClean®-htp 96 Well PCR Clean-Up Kit (MO BIO Laboratories, Carlsbad, CA, USA) according to the manufacturer's instructions with one exception: amplicons were eluted in 125µL Elution Buffer. Specimens' purified DNA content was quantified using the Quant-iT™ High-Sensitivity dsDNA Assay Kit (Invitrogen, Carlsbad, CA, USA), and 82 ng DNA per specimen was pooled into a single tube. Specimens whose amplicon DNA concentrations were insufficient to be pooled in equimolar concentrations were pooled using their entire available volume, approximately 100-115µL. Pooled amplicons were then ethanol precipitated and resuspended in 400µL pH 8.0 TE buffer (Sigma). Resuspended amplicons were than size-selected using agarose gel electrophoresis, and recovered using the QIAquick Gel Extraction Kit (Qiagen, Valencia, CA, USA) according to manufacturer's instructions. Two aliquots of 50µL amplicon were separately sequenced on an Illumina MiSeq v3 flowcell by the W.M. Keck Center for Comparative Functional Genomics at the University of Illinois, Urbana-Champaign, USA.

**Negative control description and processing**

Negative controls consisted of two types: reagent-only and blank-swab controls. Reagent-only controls consisted of empty PowerSoil® wells to which all DNA extraction, PCR, and sequencing reagents were added. One reagent-only control was included for each of six plates in either well B2 or B11. Blank-swab controls consisted of unopened foam swabs from the same lot as those used for sample collection. Control swabs were not distributed to study subjects prior to use. Four blank-swab controls were loaded into each of six PowerSoil® plates when true samples were loaded, into either wells G1,G2,H1,H2, or wells G11,G12,H11,H12.

# Supplemental Figures

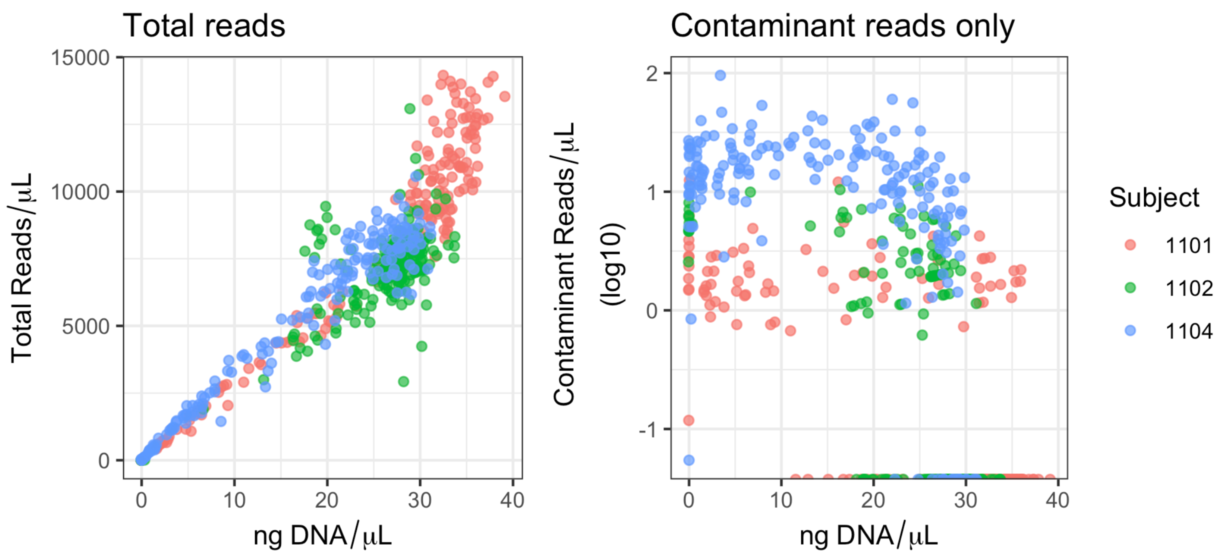


**Figure S1. Concentration of contaminant features across oral samples.** ASVs from the oral dataset were classified using decontam's prevalence method at the default threshold 0.1. Concentrations of total (left) or contaminant (right) DNA (in reads per microliter of sample added to the DNA sequencing pool) are plotted against the total post-PCR DNA concentration of the sample. Samples are colored by research subject. The total concentration of contaminants is roughly even across samples, and independent of DNA concentration.

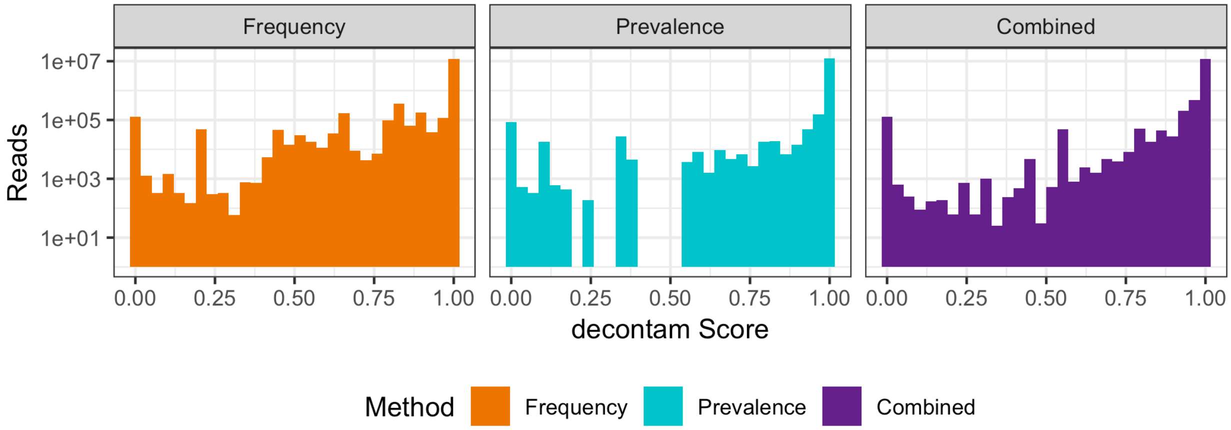


**Figure S2. Histogram of decontam scores in the oral mucosa dataset, weighted by ASV relative abundance.** Scores for each amplicon sequence variant (ASV) present in two or more samples were computed by the frequency, prevalence and combined methods as implemented in the *isContaminant* function in the decontam R package. The histogram of scores for each method is shown, after weighting by the number of reads of each ASV. The y-axis is log-scaled. Most reads are assigned high scores, indicating non-contaminant origin.

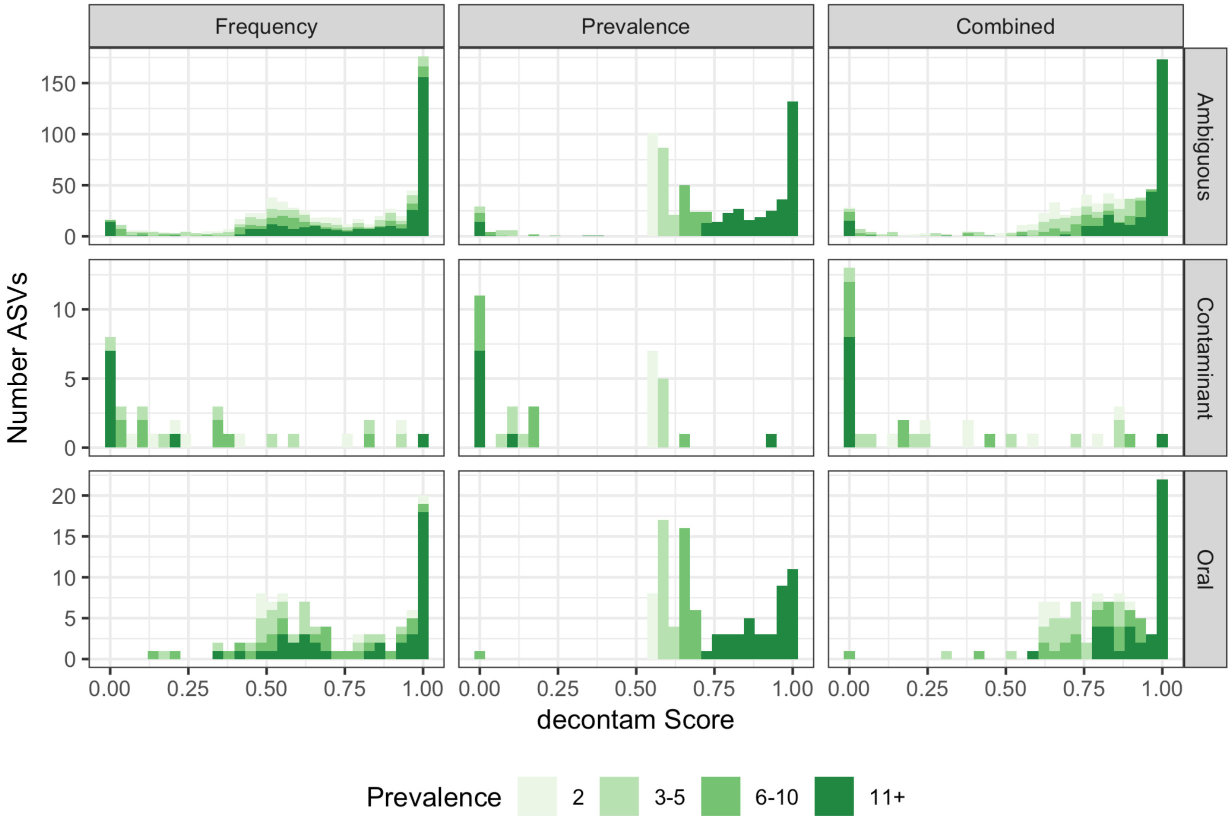


**Figure S3. Histogram of decontam scores in the oral mucosa dataset, stratified by reference-based classification.** Scores for each amplicon sequence variant (ASV) present in two or more samples were computed by the frequency, prevalence and combined methods as implemented in the *isContaminant* function in the decontam R package. The histogram of scores is shown, with color intensity depending on the number of samples (or prevalence) in which each ASV was present. Separate histograms are shown for ASVs classified as Ambiguous, Contaminant and Oral sequences based on comparison to curated reference databases (Methods). Reference-based contaminants are predominantly assigned low scores, reference-based oral ASVs are predominantly assigned high scores, while ambiguous ASVs consist of a mixture of high and low scores.

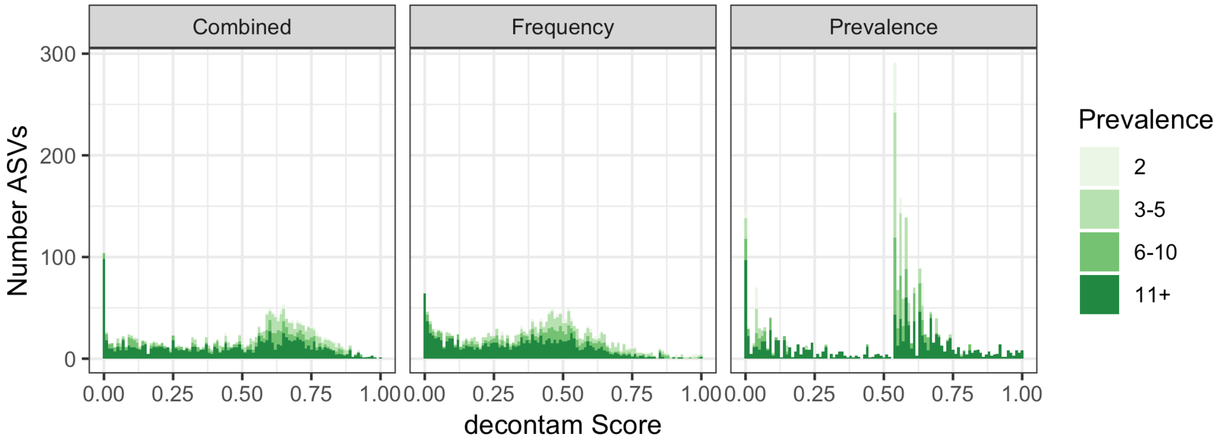


**Figure S4. Histogram of decontam scores in the preterm birth dataset.** Scores for each amplicon sequence variant (ASV) present in two or more samples were computed by the frequency, prevalence and combined methods as implemented in the *isContaminant* function in the decontam R package. The histogram of scores is shown, with color intensity depending on the number of samples (or prevalence) in which each ASV was present. A sharp peak in the score histogram is evidence at around P = 0.1.

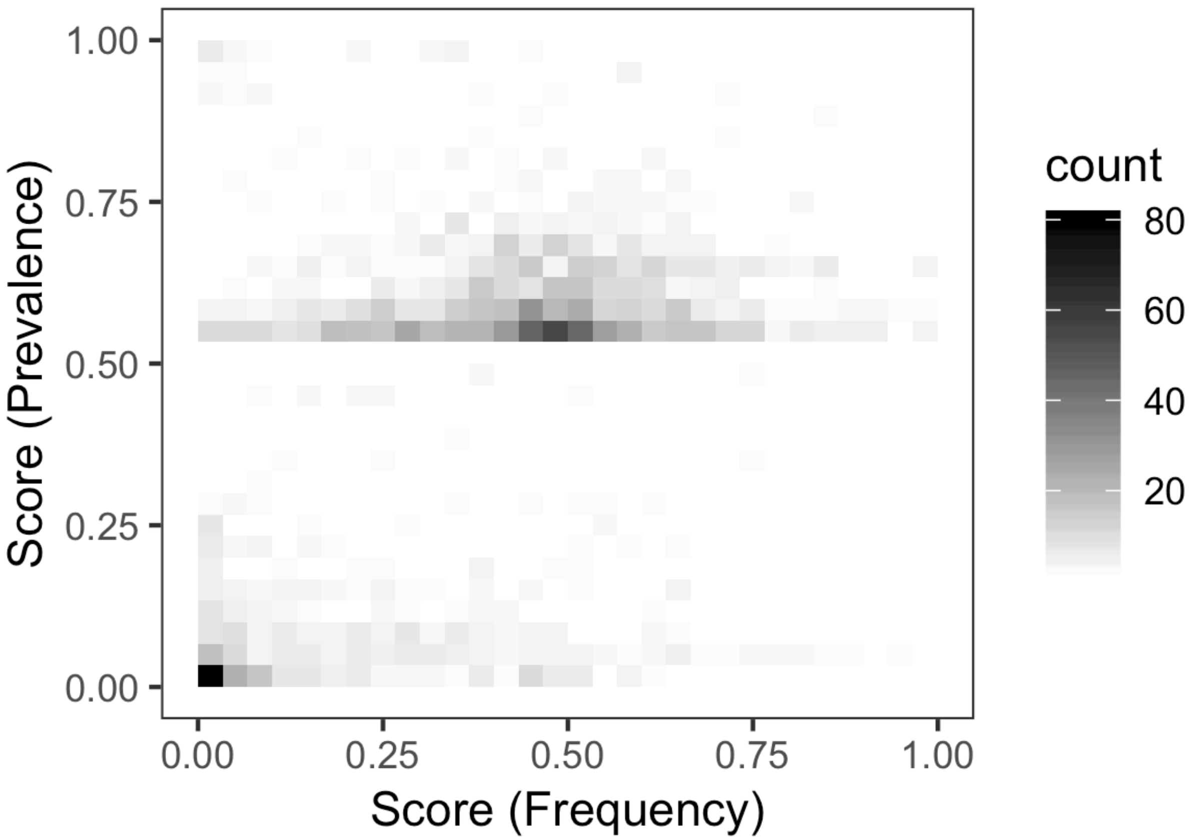


**Figure S5. Density plot of scores assigned by the frequency and prevalence methods in the preterm birth dataset.** Scores for each amplicon sequence variant (ASV) present in two or more samples were computed by the frequency and prevalence methods as implemented in the *isContaminant* function in the decontam R package. The density of joint score assignments is shown, with dark colors indicating higher numbers of ASVs assigned scores corresponding to that x-y position. Scores assigned by the frequency and prevalent methods are fairly consistent, especially at low values.

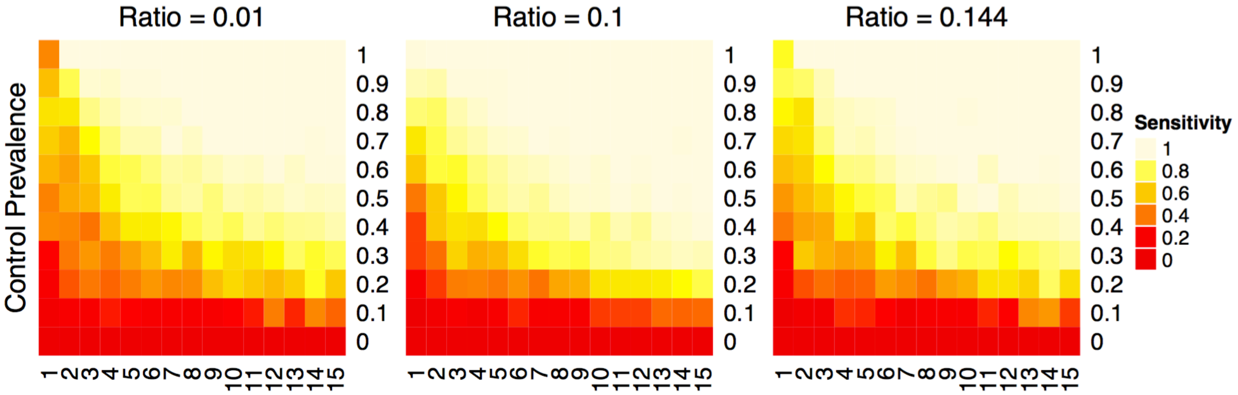


**Figure S6. Simulation analysis of prevalence method sensitivity.** Simulated prevalence distributions were generated based on a varying prevalence of contaminants in negative controls, a varying number of negative control samples, and three sample:control prevalence ratios of 0.01, 0.1, and 0.144. The mean prevalence ratio of contaminants in the oral data is 0.144. The number of controls is shown on the x-axis, and the prevalence of contaminants in negative controls on the y-axis. Sensitivity over 100 replicate simulations is shown by the color-scale. The number of true samples was fixed to 60 in these simulations, but its precise value has little impact on sensitivity as long as it is much greater than the number of controls. 5-6 negative control samples is sufficient to detect most contaminants under these scenarios.
